# Professor Campbell’s Claim

**Published:** 1857-05

**Authors:** 


### PROFESSOR CAMPBELL'S CLAIM.

We find in the last number of the Southern Medical and Surgical Journal, of Augusta, Ga., a claim of priority in the discovery of, and also the *naming* of the excito-secretory system of nerves, by Henry Fraser Campbell, M. D., of Augusta, Ga., U. S. A., member of the American Medical Association, Professor of Comparative Anatomy, &c., in the Medical College of Georgia, and senior Editor of the Southern Medical and Surgical Journal, in the form of a letter to Dr. Marshall Hall, of London, who has announced in the London Lancet, for March 1857, a system of excito-secretory nerves, as a discovery of his own, which Dr. Campbell contends was developed, and named by himself in 1850 and 1853. We regret that, on account of incessant engagements, we have not been able to give Dr. Campbell's paper the attention which we understand it deserves; we hope, however, to recur to the subject at some future time. Dr. Campbell has a high reputation for science and gentlemanly bearing, and would hardly undertake to establish a claim which was not well founded.

